# Influence of Basement Membrane Proteins and Endothelial Cell-Derived Factors on the Morphology of Human Fetal-Derived Astrocytes in 2D

**DOI:** 10.1371/journal.pone.0092165

**Published:** 2014-03-19

**Authors:** Amanda F. Levy, Maya Zayats, Hugo Guerrero-Cazares, Alfredo Quiñones-Hinojosa, Peter C. Searson

**Affiliations:** 1 Department of Materials Science and Engineering, Johns Hopkins University, Baltimore, Maryland, United States of America; 2 Institute for Nanobiotechnology, Johns Hopkins University, Baltimore, Maryland, United States of America; 3 Department of Neurosurgery and Oncology, Johns Hopkins University School of Medicine, Johns Hopkins University, Baltimore, Maryland, United States of America; Biological Research Centre of the Hungarian Academy of Sciences, Hungary

## Abstract

Astrocytes are the most prevalent type of glial cell in the brain, participating in a variety of diverse functions from regulating cerebral blood flow to controlling synapse formation. Astrocytes and astrocyte-conditioned media are widely used in models of the blood-brain barrier (BBB), however, very little is known about astrocyte culture in 2D. To test the hypothesis that surface coating and soluble factors influence astrocyte morphology in 2D, we quantitatively analyzed the morphology of human fetal derived astrocytes on glass, matrigel, fibronectin, collagen IV, and collagen I, and after the addition soluble factors including platelet-derived growth factor (PDGF), laminin, basic fibroblast growth factor (bFGF), and leukemia inhibitory factor (LIF). Matrigel surface coatings, as well as addition of leukemia inhibitory factor (LIF) to the media, were found to have the strongest effects on 2D astrocyte morphology, and may be important in improving existing BBB models. In addition, the novel set of quantitative parameters proposed in this paper provide a test for determining the influence of compounds on astrocyte morphology, both to screen for new endothelial cell-secreted factors that influence astrocytes, and to determine in a high-throughput way which factors are important for translation to more complex, 3D BBB models.

## Introduction

The human brain is composed primarily of neurons and glia along with the cells associated with the brain vasculature. Astrocytes are the most prevalent of the glial cells, participating in a variety of diverse functions from regulating cerebral blood flow to controlling synapse formation [1]–[6]. The complex star-shaped morphology of astrocytes, with their many branched and tortuous protrusions, allows for the cells to contact multiple neurons and capillaries, regulating blood flow, enhancing blood-brain barrier tightness, and maintaining water and ion homeostasis [2]–[6].

In the human brain there are four classes of astrocytes: interlaminar, protoplasmic, fibrous, and varicose projection astrocytes. All express glial fibrillary acidic protein (GFAP) and are found in different layers of the cerebral cortex [7]. Protoplasmic astrocytes, the most common type in the brain, have specialized end-feet that interact with capillaries and neurons in the brain’s gray matter. Human astrocytes are generally larger and more complex that in rodents. For example, protoplasmic astrocytes in humans are about 150 μm in diameter and have about 40 main processes emanating from the cell body [5]. In contrast, mouse protoplasmic astrocytes have an average diameter of about 60 μm and about 5 main processes [5].

Astrocytes in the human brain interact with endothelial cells through end-feet that almost completely cover the entire capillary surface [5]. The basement membrane surrounding the endothelial cells and separating the capillaries from the astrocytes is comprised of laminin and collagen type IV along with other proteins such as fibronectin and perlecan [8]–[10]. Astrocytes participate in blood-brain barrier function by increasing tight junction formation, the expression and polarization of transporters, and specialized enzyme systems [2], [5], [11], [12].

Although many of the details of astrocyte regulation of the blood-brain barrier remain to be elucidated, astrocytes and astrocyte-conditioned media are widely used in 2D cell culture models of the blood-brain barrier [13], [14]. *In vitro* permeability measurements typically involve seeding a confluent monolayer of brain capillary endothelial cells on top of a permeable transwell support with astrocytes, and sometimes pericytes, cultured in 2D on the opposite side of the membrane [15]–[18]. The morphology of these astrocytes, however, is rarely shown or discussed. Since astrocytes rarely display an astrocytic phenotype in 2D culture, this is a potential issue contributing to the lack of BBB characteristics (high electrical resistance and low permeability) of most transwell-based BBB models.

Blood-brain barrier models generally use rodent-derived astrocytes. However, in 2D cell culture, the fraction of cells exhibiting an astrocyte-like morphology with processes extending from a rounded cell body is very low, although endothelial derived factors are known to play a role in inducing an astrocytic morphology [19]–[22]. The morphology of protoplasmic astrocytes in the human brain is significantly different that in the mouse brain [5], however, very little is known about human astrocytes and their morphology in cell culture.

Here we report on the morphology of human fetal-derived astrocytes in 2D cell culture. To quantitatively determine the influence of basement membrane proteins and soluble factors on astrocyte morphology in 2D cell culture, we analyzed the morphology of cells on various physiologically relevant coatings, including matrigel, fibronectin, collagen IV, and collagen I, and after the addition soluble factors including platelet-derived growth factor (PDGF), laminin, basic fibroblast growth factor (bFGF), and leukemia inhibitory factor (LIF). We show that matrigel coating significantly increases the fraction of astrocytic cells and increases the degree of branching, and the number of branch points. Laminin, bFGF, and LIF result in a significant increase in the fraction of cells with astrocytic morphology. Co-culture of astrocytes on a confluent monolayer of brain microvascular endothelial cells results in a very large fraction of astrocytic cells with more protrusions and higher tortuosity.

## Materials and Methods

### Astrocyte and Endothelial Cell Culture

Following approval by the Johns Hopkins University Institutional Review Board, primary cultures of human fetal-derived astrocytes were obtained as described previously [23], [24]. Briefly, intraoperative human central nervous system (CNS) tissues, gestational weeks 19–21, which were obtained following written informed consent for clinical procedures, were used for this research since they were considered to be pathological waste. Tissue was mechanically dissociated and cells number and viability was determined by trypan blue incorporation. Neural cells were first cultured as neurospheres in suspension in low adherent flasks using DMEM/F12 (Sigma) medium with 2% B27 supplement, 1% penicillin-streptomycin (Invitrogen), 20 ng mL^−1^ of EGF (Peprotech) and bFGF (Peprotech), 10 ng mL^−1^ of LIF (Millipore), and 5 μg mL^−1^ of heparin (Sigma). To induce the formation of astrocytes, neurospheres were mechanically dissociated and single cells were plated on tissue culture flasks in DMEM/f12 medium (Sigma) supplemented with 10% fetal bovine serum (Sigma) and 1% penicillin-streptomycin (Invitrogen). Human brain microvascular endothelial cells (HBMECs) were isolated from an adult brain and immortalized by transfection with SV40 [25]. While immortalized human brain endothelial cells typically do not form tight junctions with low permeability and high transendothelial resistance, they have the advantages of being widely available and commonly used cells in BBB models. A recent study showed that the HBMEC cell line used in these experiments has the highest TEER values, and expresses ZO-1, VE-cadherin, and claudin-5 [26]. In addition, these cells express basement membrane proteins relevant for this study, including fibronectin, laminin, and collagen IV.

### Extracellular Matrix Protein Coatings

Nunc Lab Tek II 2-well chamber slides (0.7 cm^2^) were coated in the following extracellular matrix (ECM) components: fibronectin, collagen IV, matrigel (phenol red-free, growth factor reduced), and collagen I. Fibronectin, collagen IV, and matrigel (BD Biosciences) were diluted to concentrations of 100 μg mL^−1^ in phosphate buffered saline (PBS, Invitrogen). Wells were coated with 100 μL cm^−2^ diluted ECM proteins for 2 hours at 4°C. Wells were washed once with PBS before cell seeding. Rat tail collagen I (BD) was diluted to a concentration of 100 μg mL^−1^ in 0.02 M acetic acid. Wells were coated for 1 hour at 37°C and washed three times with PBS. Uncoated glass was used as a control. Astrocytes were seeded on the coated dishes at a concentration of 5,000 cells cm^−2^. Low magnification images of cells on different surface coatings are shown in Figure S1. For the co-culture experiments, HBMECs were seeded onto 2-well chamber slides at a concentration of 50,000 cells cm^−2^. Cells were grown to confluence for 48 hours in M199 media. After confluence was reached, the medium was replaced with DMEM/f12 and astrocytes were seeded on top of the monolayer at a density of 5,000 cells cm^−2^. In all experiments, astrocytes were fixed after 24 hours. All experiments on coated surfaces were performed in triplicate.

### Soluble Factors

Astrocytes were seeded at a concentration of 5000 cells cm^−2^ onto 2-well chamber slides. Cell culture media was supplemented with 10 ng mL^−1^ platelet-derived growth factor (PDGF) (Sigma P4056), laminin (Sigma, L2020), basic fibroblast growth factor (bFGF) (Millipore, GF003), or leukemia inhibitory factor (LIF) (Sigma, L5283). Unsupplemented astrocyte media was used as a control.

### Immunofluorescence Microscopy and Image Analysis

Cells were fixed in 3.7% formaldehyde after 24 hours of culture. After fixing for 10 minutes at room temperature, cells were washed with PBS three times and permeabilized with 0.01% Triton-X 100 (Sigma) for 5 minutes. Cells were then washed with PBS and blocked with 10% donkey serum (Millipore) in PBS for 30 minutes at room temperature. Cells were then incubated with primary antibodies in 10% donkey serum overnight at 4°C. Goat anti-glial fibrillary acidic protein (aGFAP, Santa Cruz) was used at a dilution of 1∶50 to label astrocytes. Rabbit anti-zona occludens-1 (ZO-1, BD) was used at a dilution of 1∶200 to label endothelial cell tight junctions. Cells were washed with PBS three times for five minutes each following primary antibody incubation, and then incubated with secondary antibodies in 10% donkey serum for 1 hour at room temperature. Donkey anti-goat IgG 488 and donkey anti-rabbit IgG 568 (Invitrogen) were used to fluorescently label the GFAP and ZO-1, respectively, and DAPI was used to stain nuclei. Cells were washed with PBS three times before imaging.

Fluorescence images were acquired with a Nikon TiE microscope equipped with a Photometrics camera using NIS elements software. Experiments were designed such that the density of adherent cells was sufficiently low for single cell imaging. Images were recorded of adherent cells at random locations in the wells. For analysis of the number of astrocytic and non-astrocytic cells, all recorded images, including those with multiple cells, were included. For detailed morphological analysis of astrocytic cells expressing protrusions, only images of isolated cells were analyzed. Cells expressing astrocyte-like processes were traced in ImageJ and analyzed for the following morphological parameters: number of primary, secondary, and tertiary protrusions emanating from the main cell body, cell body area, total protrusion length, the overall size (defined by the diameter of the smallest circle that can enclose the cell and all of its processes), the number of branch points (the sum of secondary and other higher order branches), and the degree of branching (number of branch points divided by the number of primary protrusions).

## Results and Discussion

### Influence of Basement Membrane Proteins on Astrocyte Morphology

**Figure 1:**
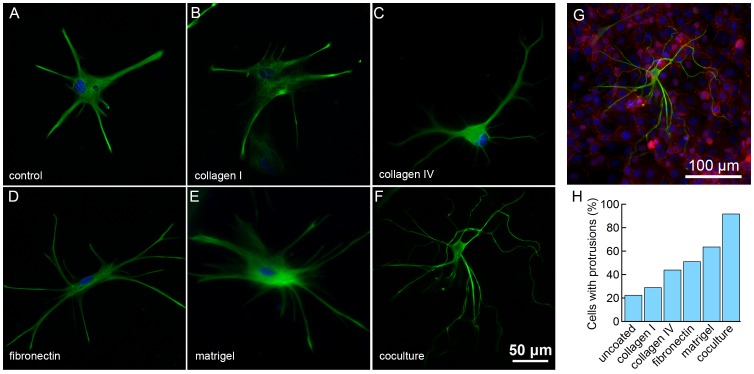
Influence of ECM coating on astrocyte morphology after 24 hours. Fluorescence images of astrocytes stained for GFAP (green) and DAPI (blue) on (A) glass, (B) collagen I, (C) collagen IV, (D) fibronectin, (E) matrigel, and (F) co-culture on a confluent monolayer of HBMECs. (G) Astrocyte (from panel (F)) seeded on a confluent monolayer of HBMECs, stained for GFAP (green), DAPI (blue), and ZO-1 (red). (H) The percentage of cells with protrusions. Total number of cells analyzed: uncoated (N = 103), collagen I (N = 85), collagen IV (N = 61), fibronectin (N = 63), matrigel (N = 54), co-culture (N = 58).

To assess the role of basement membrane proteins on the morphology of human fetal-derived astrocytes, cells were seeded on various physiologically relevant coatings (Figure 1). Only 23% of astrocytes seeded on glass exhibited astrocyte-like morphology. Rat tail collagen I, which is largely absent in the smaller vessels of the brain, resulted in 29% of cells with processes, a small increase in compared to the control. Collagen IV (44%) and fibronectin (51%), both present in the basement membrane at the BBB, resulted in a further increase in the number of astrocytic cells compared to the controls. However, when seeded on matrigel prepared from purified mouse basement membrane, the fraction of astrocytic cells increased to 64%. For comparison astrocytes were also seeded on confluent monolayers of HBMECs (Figure 1G), resulting in 92% of the cells with astrocyte-like protrusions. These results (see Table S1 for summary) indicate that basement membrane proteins and endothelial cells have a strong influence on the formation of astrocytic processes in 2D cell culture.

All of the cells expressing astrocyte-like processes were analyzed for the following morphological parameters: number of primary, secondary, and tertiary protrusions emanating from the main cell body, cell body area, total protrusion length, the overall size, the number of branch points, and the degree of branching. Although the number of branch points and the degree of branching are parameters derived from the number of primary, secondary, and tertiary protrusions, they provide complementary information and are useful in comparison to the literature. In addition, the protrusion tortuosity (τ) was determined from τ = *l*/c where *l* is the arc length of the processes and c is the shortest end-to-end distance.

**Figure 2:**
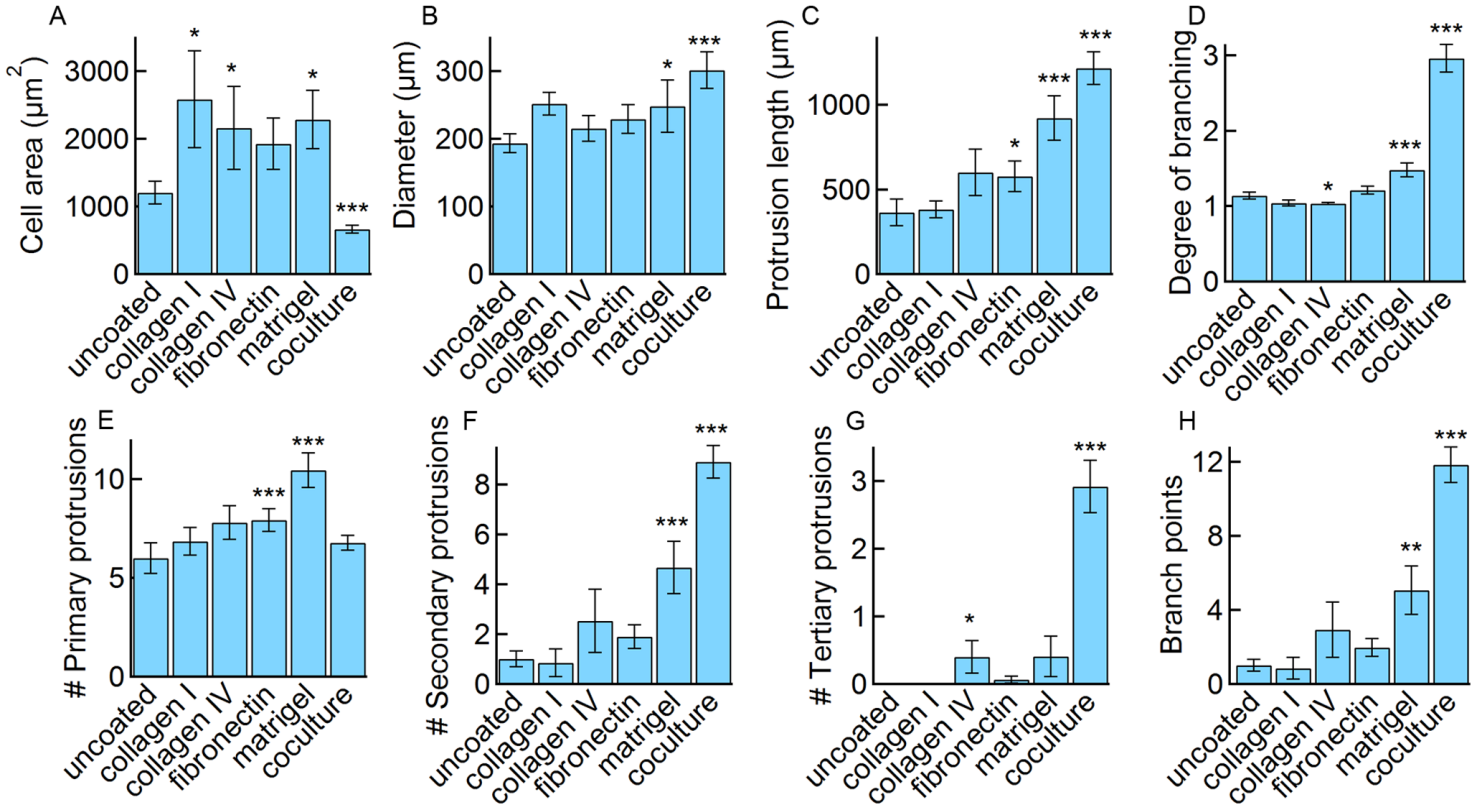
Influence of surface coatings on astrocyte morphology. (A) The cell area defined by the area of the cell body. (B) the cell diameter is overall size defined by the diameter of the smallest circle that can enclose the cell and all of its processes. (C) The protrusion length is the total length of all protrusions. (D) The degree of branching is the number of branch points divided by the number of primary protrusions. (E) The number of primary protrusions represents the number of protrusions emanating from the cell body. (F) The number of secondary protrusions represents protrusions emanating from primary protrusions. (G) The number of tertiary protrusions represents protrusions emanating from secondary protrusions. (H) The number of branch points represents the sum of secondary and other higher order protrusions (equivalent to the number of bifurcations). Data represent mean ± SE. Statistical significance was determined using a student’s t-test test. ***P≤0.01, **P≤0.05, *P≤0.1. Only cells with astrocyte-like morphology were analyzed (the total number of cells and the fraction of cells with astrocyte-like morphology are provided in Figure 1).

First we summarize the results for astrocyte morphology on coated surfaces compared to the uncoated glass control (Figure 2). In general, astrocytic cells on coated surfaces exhibited 5–10 primary protrusions and an overall diameter of 200–300 μm. For astrocytes on collagen I and collagen IV, the overall cell diameter, the number of primary, secondary, and tertiary protrusions, as well as the total protrusion length were not statistically different from the uncoated controls. The morphology of astrocytes on fibronectin was also similar to the uncoated controls for most parameters. In contrast, for astrocytes seeded on matrigel, the number of primary (10.4±0.9) and secondary (4.7±1.1) protrusions, the overall protrusion length (921±130 μm), the number of branch points (5.1±1.3), and the degree of branching (1.5±0.1) were significantly higher than the control (6.0±0.8 primary and 1.0±0.3 secondary protrusions, 365±79 μm total protrusion length, 1.0±0.3 branch points, and a degree of branching of 1.1±0.1). These results suggest that matrigel plays a significant role in eliciting astrocyte morphology in 2D.

The overall diameter of astrocytic cells on basement membrane proteins or a confluent monolayer of endothelial cells (200–300 μm) is larger than for astrocytes in the human brain (about 150 μm), whereas the number of primary protrusions (5–10) is lower (about 40) [5]. These results are not surprising since the human fetal derived astrocytes are confined to two-dimensional growth. However, the size and number of processes are significantly larger than for astrocytes in the mouse brain where the average size is about 60 μm and there are about 5 main processes [5].

Co-culture of human fetal-derived astrocytes on confluent monolayers of brain microvascular endothelial cells was found to dramatically enhance astrocyte morphology over controls (Figures 1 and 2). Astrocytes cultured on endothelial cells exhibited a similar number of primary protrusions (6.8±0.8), compared to the uncoated controls (6.0±0.4), but a significant increase in the number of secondary (8.9±0.66) and tertiary (2.9±0.38) protrusions compared to the control (1.0±0.31 secondary and 0 tertiary protrusions), resulting in a large number of branch points (11.8±1.0) and a high degree of branching (3.0±0.2) compared to the control (1.0±0.3 branch points and a degree of branching of 1.1±0.1). The cell body area (661±55 μm^2^) and the overall diameter (301±27 μm) were smaller than the uncoated control (1200±171 μm^2^ and 193±14 μm, respectively). The small cell body area may be related to the localization of expression of ECM proteins to the cell-cell junctions in the HBMEC monolayer. In addition, the processes often followed cell-cell boundaries, implicating ECM proteins and possibly soluble factors secreted by the endothelial cells in influencing astrocyte morphology. Brain capillary endothelial cells are known to produce fibronectin, laminin, and collagen IV in culture [8].

**Figure 3:**
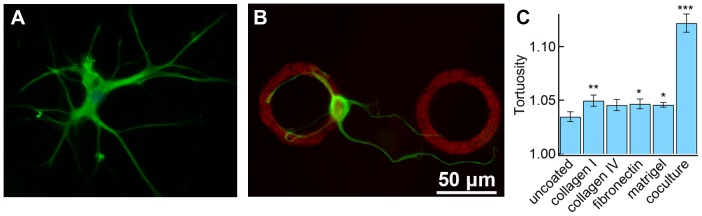
Tortuosity of astrocytes. (A) Immunofluorescence image of an astrocyte on HBMEC-derived ECM. Endothelial cells were grown to confluence on glass-bottom dishes, and removed from the dish with a lysis buffer containing 0.5% Triton X-100 and 20 mM NH_4_OH in PBS. (B) Immunofluorescence image of an astrocyte on 50 μm inner diameter fibronectin rings. Astrocytes tended to trace the rings and have smaller cell bodies like those seen in co-culture. Fibronectin (red), GFAP (green). (C) Tortuosity of astrocytes on surface coatings and in co-culture. The tortuosity (τ) is given by τ = *l*/c where *l* is the arc length of the processes and c is the shortest end-to-end distance. For a straight line τ = 1, whereas for a circle τ = ∞. While all of the surface coatings result in very small increases over the control, the astrocytes in co-culture have significantly higher tortuosities. Statistical significance was determined using a student’s t-test. ***P≤0.01, **P≤0.05, *P≤0.1.

The important role of ECM proteins was further confirmed by seeding astrocytes on patterned surfaces. Astrocytes seeded on HBMEC-derived ECM after lysis of a confluent monolayer of HBMECs (Figure 3A) showed cells with smaller cell bodies, and branched, tortuous processes, similar to the morphology of astrocytes seeded on HBMEC monolayers (Figure 1G). Astrocytes on micropatterned fibronectin rings (Figure 3B) showed similar features, where many processes were seen to follow the ring patterns, and the astrocyte cell bodies tended to be much smaller than on the uniformly fibronectin-coated glass.

In contrast to cells seeded on glass coated with basement membrane proteins, cells seeded on a confluent monolayer of endothelial cells showed a significant increase in all parameters except cell body area and the number of primary protrusions (Figure 2). The cell body area decreased from 1200 to 661 μm^2^ on the endothelial cell monolayer. While the number of primary protrusions was similar to cells on uncoated glass, the number of branch points and degree of branching was significantly increased and larger than on the coated glass.

Collagen I and IV, fibronectin and matrigel all promote an increase in cell body area (Figure 2A) compared to uncoated glass. In contrast, the cell body area was significantly smaller for astrocytes on a monolayer of endothelial cells (Figure 1G), on HBEMC-derived ECM (Figure 3A), or on fibronectin rings (Figure 3B). The end-feet of astrocytes in the brain are in contact with basement membrane proteins whereas the cell body is in contact with interstitial fluid and the hyaluronic acid-based extracellular matrix. Our results suggest that the lack of contact with basement membrane proteins plays a role in minimizing the cell body area in the brain. Therefore, in cell culture, surfaces patterned with basement membrane proteins or endothelial cell monolayers result in morphologies that more closely resemble astrocytes in the brain.

Primary murine astrocytes seeded on poly-lysine modified surfaces have been shown to express processes in less than 5% of cells [21]. Three days after the addition of a Rho GTPase inhibitor (the *Clostridium botulinum* C3 toxin), over 80% of these primary murine astrocytes expressed protrusions. Analysis of the astrocytic cells revealed that the total protrusion length increased from about 300 μm to about 800 μm and the number of branch points increased from about 1 to about 8 [21]. The total protrusion length of human fetal-derived astrocytes on matrigel (920 μm) or co-culture (1200 μm), and the number of branch points (5.1 on matrigel and 11.8 on co-culture) are similar (Figure 2), suggesting that the Rho GTPase inhibitor may modulate the same pathways as the factors secreted by the HBMECs.

The straightness of the protrusions was assessed by measuring the tortuosity, τ = *l*/c. For a straight line τ = 1, whereas for a circle τ = ∞. The tortuosity of the processes was slightly higher on fibronectin, collagen I, and matrigel compared to the uncoated surface. However, the tortuosity was significantly higher in co-culture where the processes tended to follow the cell-cell boundaries.

### Influence of Endothelial Cell-expressed Soluble Factors on Astrocyte Morphology

To elucidate the role of soluble factors expressed by endothelial cells, human fetal-derived astrocytes seeded on uncoated glass were incubated with platelet-derived growth factor (PDGF), laminin, basic fibroblast growth factor (bFGF), or leukemia inhibitory factor (LIF), for 24 hours to determine their influence on astrocyte morphology. PDGF is expressed in brain capillaries and, in addition to recruiting pericytes to the BBB [27], promotes division and inhibits differentiation of glial cells from the rat optical nerve [28]. Laminin is an extracellular matrix protein present in the basement membrane and is known to promote neurite outgrowth *in vitro* [29]. bFGF is expressed by both astrocytes and endothelial cells at the BBB. LIF is expressed by endothelial cells and has been shown to induce astrocyte differentiation [22]. While the mechanism of LIF-induced astrocyte differentiation is not well characterized, it is thought to involve glycoprotein 130 dependent signaling pathways [30].

**Figure 4:**
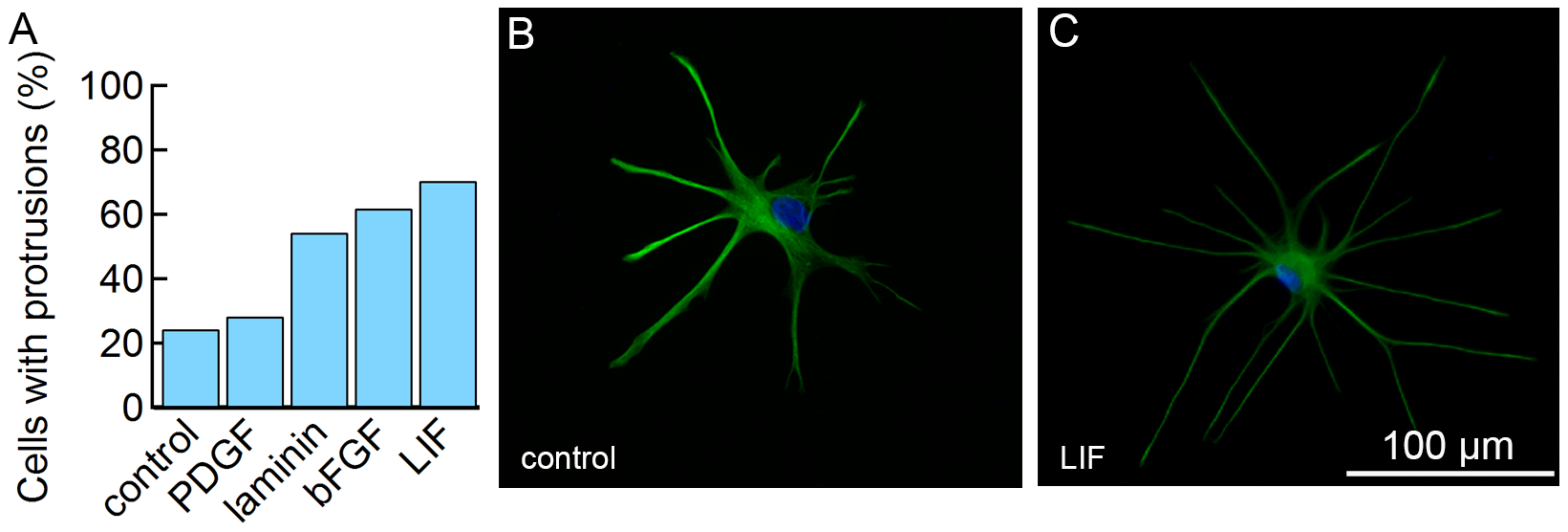
Morphological analysis of astrocytes cultured on glass with the additional of soluble factors. (A) Percentage of cells with protrusions. Control (N = 79), PDGF (N = 81), laminin (N = 68), bFGF (N = 71), LIF (N = 74). Immunofluorescence images of (B) control and (C) astrocytes treated with LIF stained for GFAP (green) and DAPI (blue).

All of the soluble factors studied here increased the fraction of astrocytes with processes (Figure 4A). The addition of PDGF (28%), laminin (54%), bFGF (62%), or LIF (70%) resulted increases in the fraction of cells with astrocyte-like morphology compared to the controls (23%). Indeed, the addition of LIF to cells seeded on uncoated glass resulted in more astrocytic morphology than on matrigel (64%) (Figure 1H).

**Figure 5:**
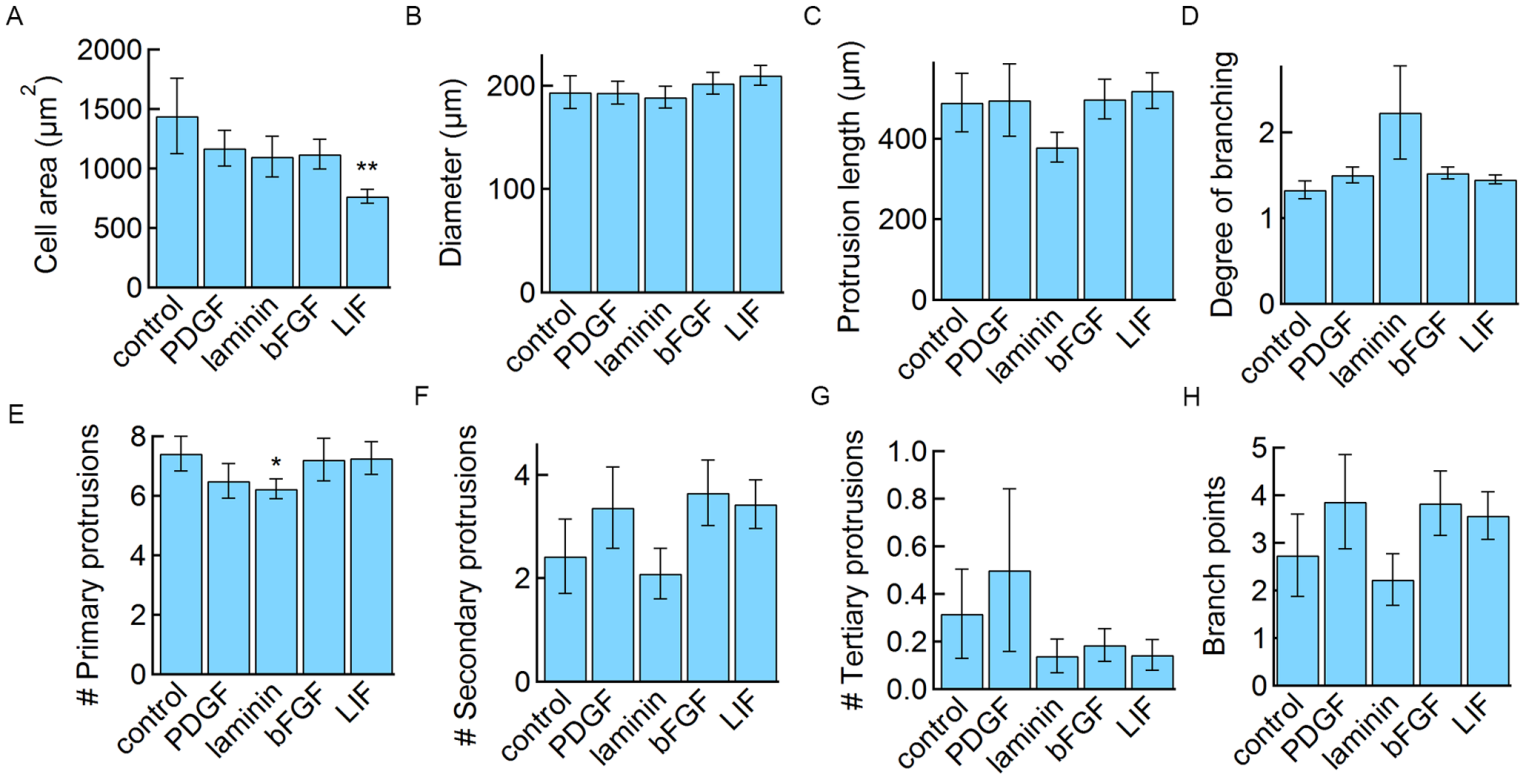
Influence of soluble factors on astrocyte morphology. Data represent mean ± SE. Statistical significance was determined using a student’s t-test. ***P≤0.01, **P≤0.05, *P≤0.1. See Figure 4 for the number of cells analyzed.

The addition of these soluble factors did not result in any systematic differences in cell morphology (Figure 5). However, LIF had a statistically significant effect on the cell body area (767±59 μm^2^) (Figure 5A), which was smaller than the control media (1440±316 μm^2^), and similar to the cell body area observed in co-culture (660±55 μm^2^) (Figure 2A).

Both co-culture with vascular endothelial cells and the addition of LIF have been shown to increase the fraction of GFAP positive rat astrocyte precursor cells, suggesting that LIF plays an important role in astrocyte induction [22]. These studies suggest that vascular endothelial cells are the source of LIF for astrocyte differentiation, however, the correlation between GFAP expression and cell morphology was not reported [22]. In a related study, the culture of primary mouse astrocytes in brain capillary endothelial cell conditioned media was found to qualitatively increase the number of cells with processes [20].

These results have implications for the design of 2D transwell models for the blood-brain barrier and more broadly on the role of brain microvascular endothelial cells in regulating astrocyte morphology and function. While the use of astrocytes in 2D models of the blood-brain barrier is very common and known to increase transendothelial electrical resistance, their morphology is usually ignored, and may be an overlooked factor contributing to the lack of reproducible protocols. Furthermore, human fetal-derived astrocytes provide a potential source of cells for blood-brain barrier models using human cells. In addition, the approach developed here could be used to assess the role of other cell-derived factors on astrocyte morphology, further advancing the understanding of astrocyte interactions at the blood-brain barrier and determining which factors are most important for translation to 3D co-culture and 3D blood-brain barrier models with human cell lines.

## Supporting Information

Figure S1 **Low magnification images of astrocytes on different surface coatings.** In all cases about 25,000 cells cm^−2^ were seeded into 0.7 cm^2^ wells for 24 hours. These images show the large diversity of astrocyte phenotypes, from fibroblast-like cells to more physiological cells expressing protrusions. While the number of adherent cells varies very little between the different surface conditions, the number of cells expressing protrusions is significantly greater on the fibronectin and matrigel-coated glass surfaces.
(TIF)

Table S1 **Tabulated summary of key findings.** Details of the differences between the various analyzed conditions and the uncoated glass controls, and percentage of cells displaying astrocytic morphology.
(TIF)
