# Supplementary figures and images for: Influence of Basement Membrane Proteins and Endothelial Cell-Derived Factors on the Morphology of Human Fetal-Derived Astrocytes in 2D
Source: PLoS One. 2014 Mar 19;9(3):e92165. doi: 10.1371/journal.pone.0092165 (PMC3960172; doi:10.1371/journal.pone.0092165)

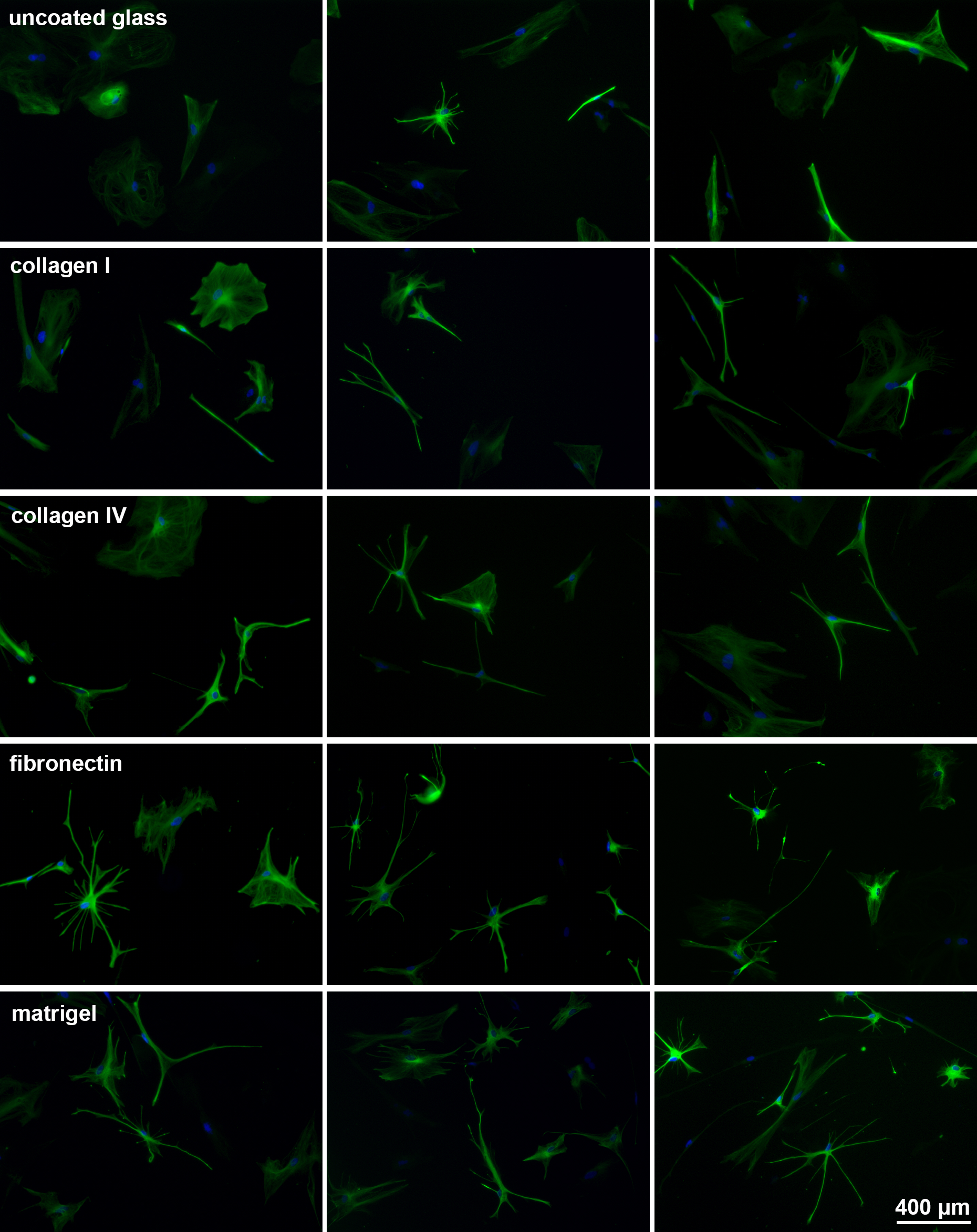

Supplement: Figure S1 — Low magnification images of astrocytes on different surface coatings. In all cases about 25,000 cells cm−2 were seeded into 0.7 cm2 wells for 24 hours. These images show the large diversity of astrocyte phenotypes, from fibroblast-like cells to more physiological cells expressing protrusions. While the number of adherent cells varies very little between the different surface conditions, the number of cells expressing protrusions is significantly greater on the fibronectin and matrigel-coated glass surfaces. (TIF) [file pone.0092165.s001.tif]

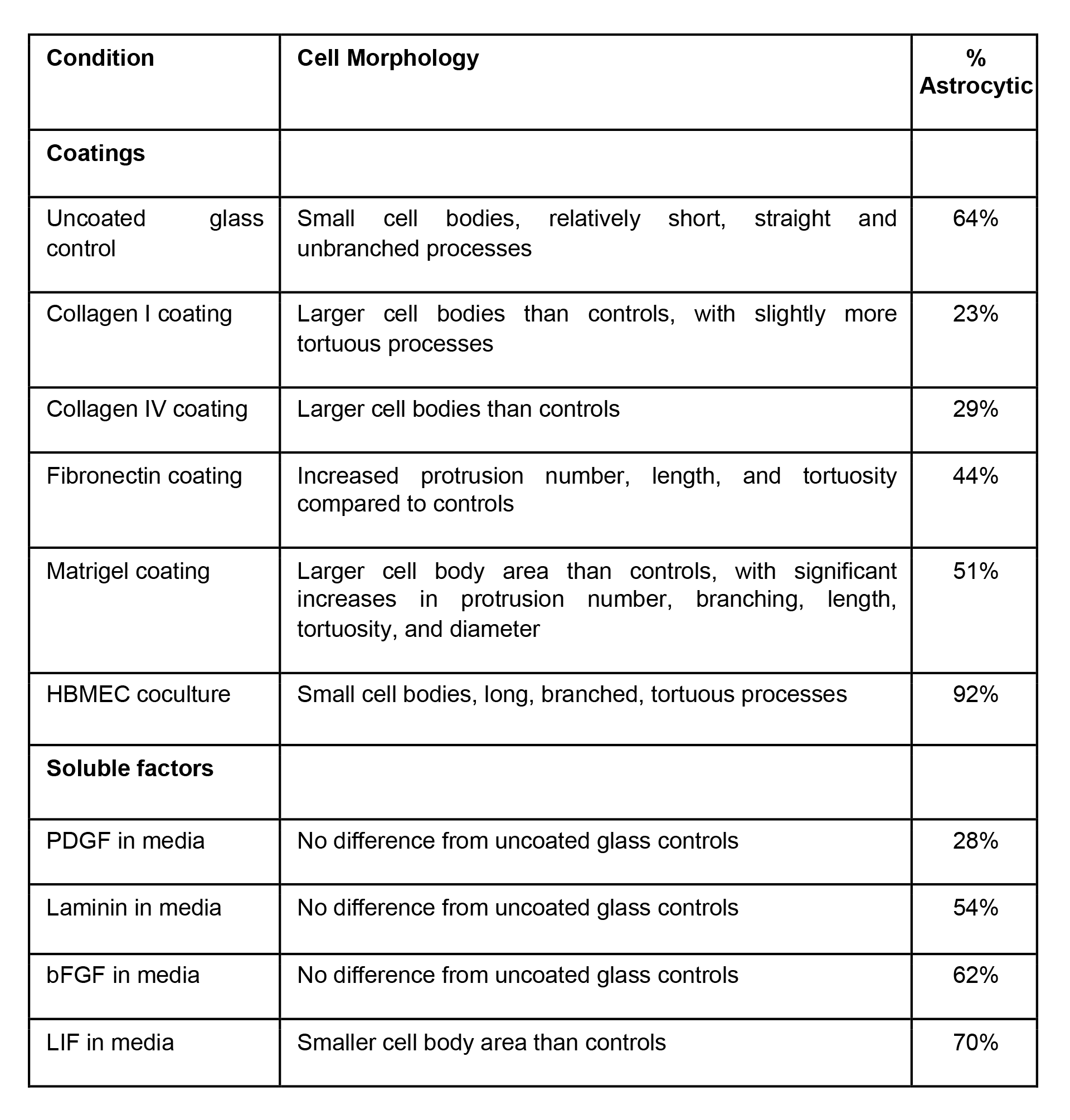

Supplement: Table S1 — Tabulated summary of key findings. Details of the differences between the various analyzed conditions and the uncoated glass controls, and percentage of cells displaying astrocytic morphology. (TIF) [file pone.0092165.s002.tif]
